# Supplementary material for: Lateral orbitofrontal cortex anticipates choices and integrates prior with current information
Source: Nat Commun. 2017 Mar 24;8:14823. doi: 10.1038/ncomms14823 (PMC5376669; doi:10.1038/ncomms14823)
Supplement: Supplementary Information — Supplementary Figures, Supplementary Methods and Supplementary References [file ncomms14823-s1.pdf]

$$p(S_n|S_{n-1},C_{n-1}) = \begin{cases} p(S_n) & if\ S_{n-1} = C_{n-1} \\ \delta(S_n - S_{n-1}) & if\ S_{n-1} \neq C_{n-1} \end{cases}$$

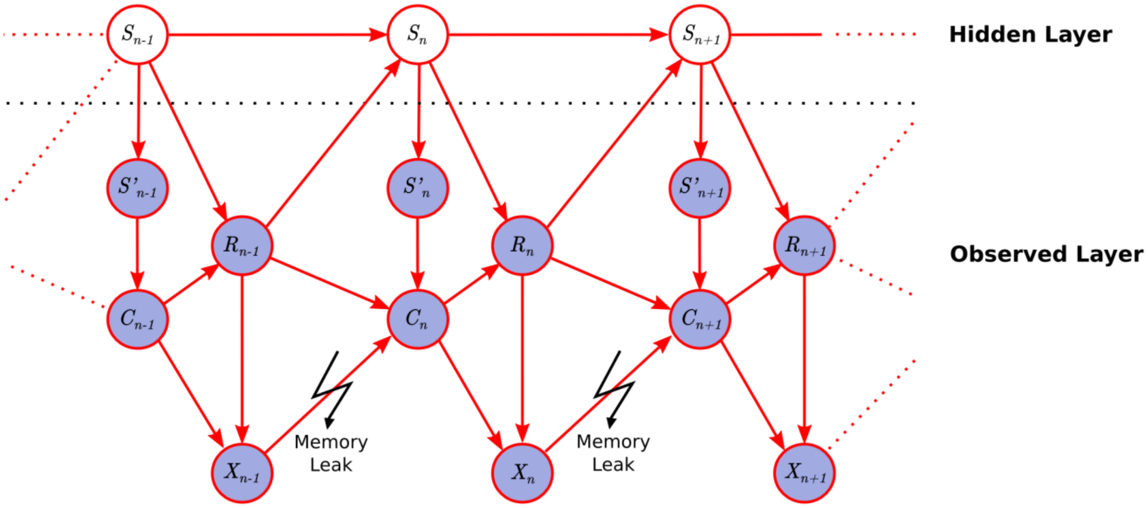

Supplementary Figure 1:

The sequence of trials follows an outcome-coupled hidden Markov chain: after a correct response a new random stimulus condition is drawn in the next trial, while after an incorrect response the same stimulus is repeated in the next trial (top equation). The hidden layer is composed by the actual stimulus sequence that is presented to the rat ($\{S_n\}$), while the observed layer comprises the set of variables that are accessible to the rat: observed stimulus (S'_n), choice (C_n), reward (R_n) and the second-order interaction between choice and reward (X_n) (labels follow the same convention as in the main text and Methods). Memory leak is incorporated into the model by allowing the choice to be based on a corrupted version of the previous second-order prior variable. The mathematical formulation of the outcome-coupled hidden Markov chain is displayed at the top.

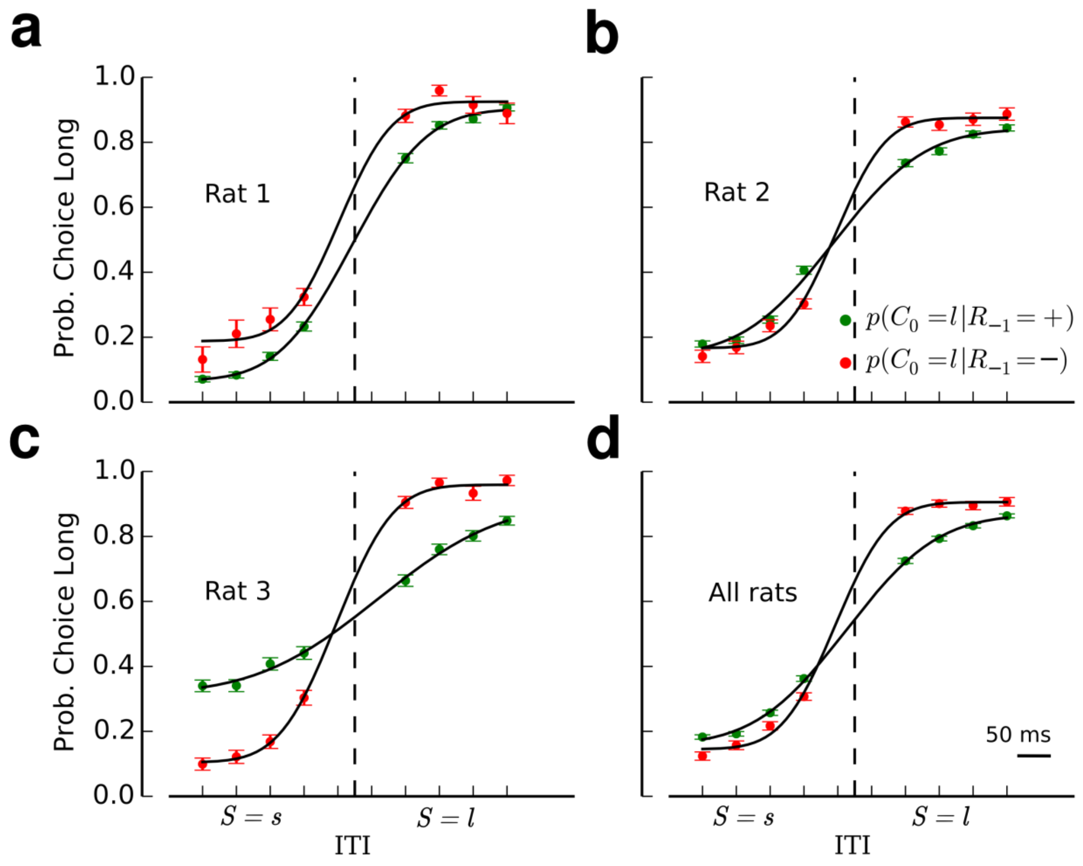

Supplementary Figure 2

Psychometric curves (probability choice long vs. ITI) after correct response (green dots) and after incorrect responses (red dots) for each rat (three first panels) and for all rats (fourth panel). Error bars are as in Fig. 1 (one standard deviation over boostrap iterations). Fits correspond to a lapse-corrected cumulative Gaussian (see Methods).

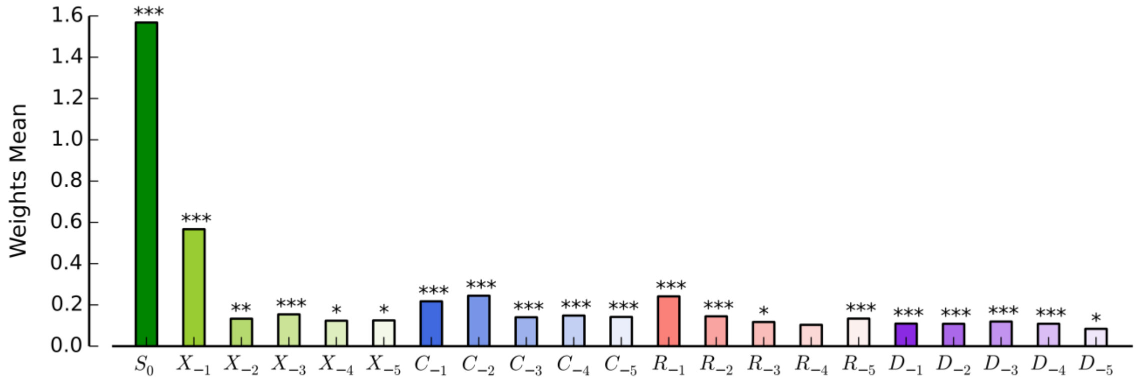

Supplementary Figure 3:

Logistic regression analysis predicting upcoming choice C_0 based on a linear combination of binary regressors (displayed along the horizontal axis) shows that stimulus and second-order prior variables are the strongest predictors for behavior. Variables from two and more trials into the past have a weaker effect on the upcoming choice. Due to the large number of trials in our datasets and because of the hidden nature of the stimulus Markov chain, their effect is in most cases still significant (evaluated using a permutation test, SupplementaryMethods), * = $p < 0.05$, ** = $p < 0.01$, *** = $p < 0.001$.

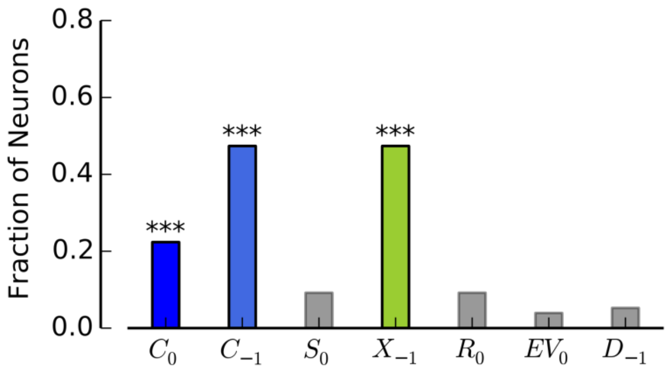

Supplementary Figure 4:

Fraction of neurons with significant regressors for each of the variables listed in the horizontal axis when the linear model was fitted exclusively using trials after correct responses. Note that in this case previous choice and second-order prior are identical variables, but they are displayed separately for better comparison with Fig. 4. One-tailed binomial test, n = 76, * = $p < 0.05$, ** = $p < 0.01$, *** = $p < 0.001$.

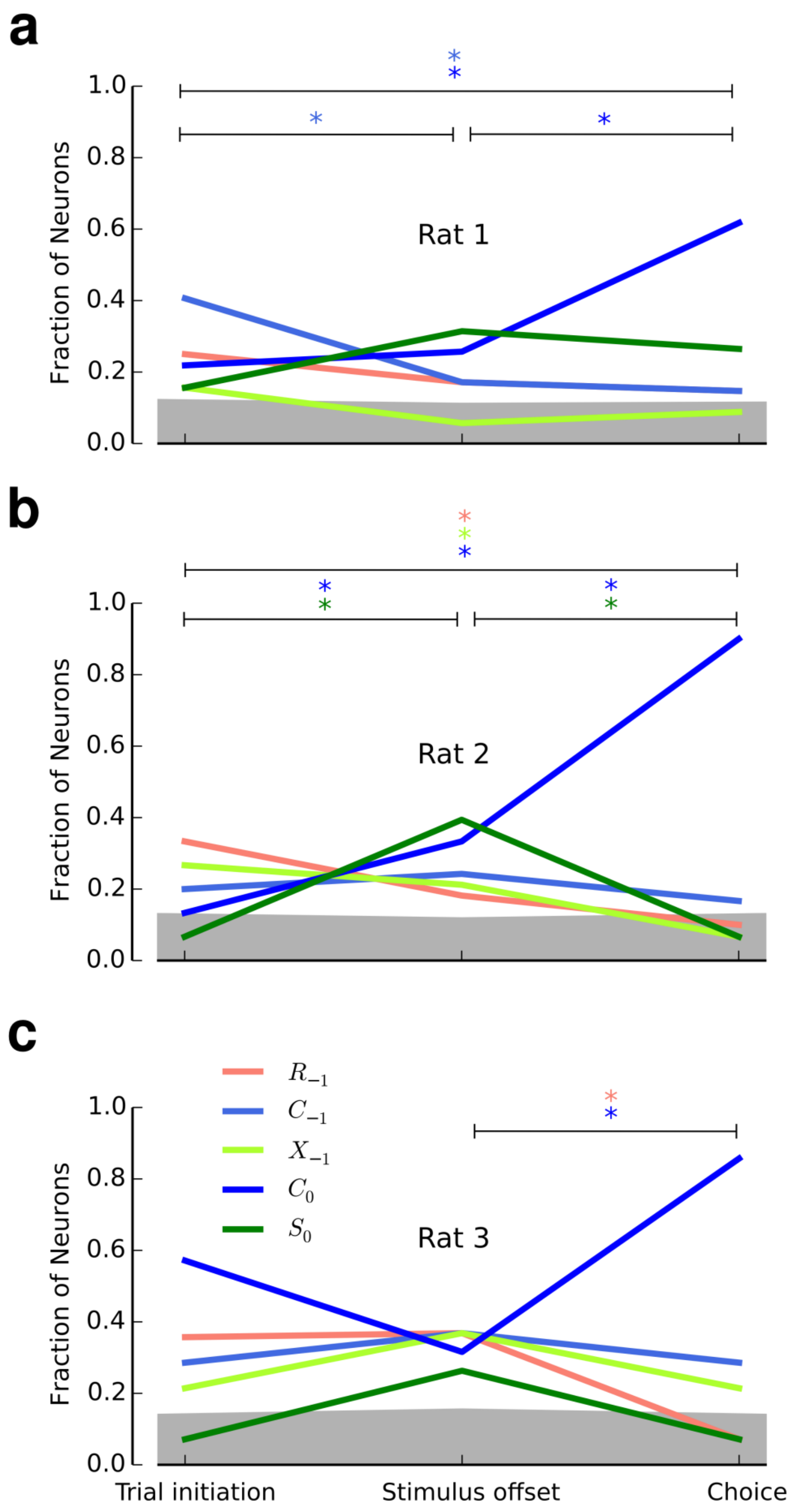
a
1.0
0.8
0.6
0.4
0.2
0.0
Fraction of Neurons
Rat 1
b
1.0
0.8
0.6
0.4
0.2
0.0
Fraction of Neurons
Rat 2
c
R_{-1}
C_{-1}
X_{-1}
C_0
S_0
1.0
0.8
0.6
0.4
0.2
0.0
Fraction of Neurons
Rat 3
Trial initiation
Stimulus offset
Choice

Supplementary Figure 5

Temporal evolution of the fractions of neurons with significant regressors for each rat (a-c). Significance of the fraction of neurons corresponds to values above the gray rectangle (p < 0.05, n = 76 (trial-initiation), n = 87 (stimulus-offset), n = 78 (choice)). Thus, for the three rats the upcoming choice, previous reward and second-order prior are significantly encoded at trial initiation, before stimulus onset (except for rat 2 for which upcoming choice is not significant at the borderline level 0.06). Consistent for the three rats, current stimulus S_0 is strongly encoded during the stimulus offset period. Significance of the differences in fractions of neurons are indicated with elongated bars at the top and a start with the same color as the color variable (* = p < 0.05; see Methods). Only significant changes are indicated (for rat 3, there are not significant changes from trial initiation to stimulus offset, likely because of noisier estimates of the fractions due to the lower number of neurons recorded in this animal). Overall, for the three rats there is a significant increase of choice-related signals across major task periods.

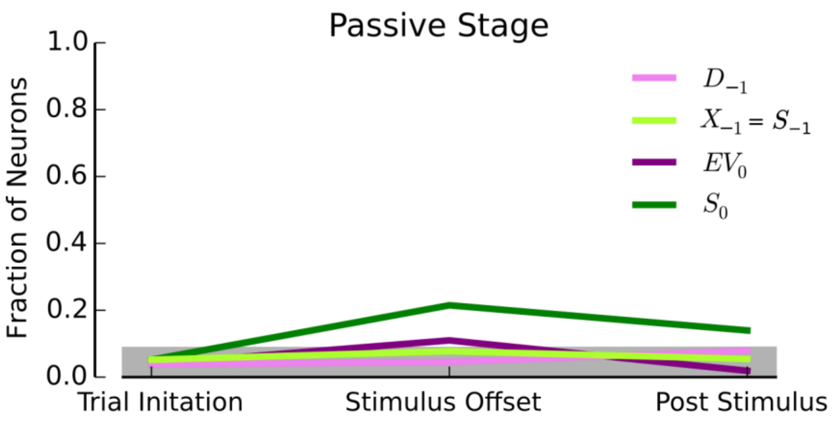

Supplementary Figure 6

Temporal evolution of the fraction of neurons with significant regressors for the stage where rats are passively exposed to the same set of stimuli as in the decision-making task. The depicted fraction of neurons corresponds to the mean of the two passive stages. Neurons exclusively represent current stimulus and current expected value (difficulty) at stimulus-offset period. Thus, during the passive stage second-order prior information is no longer encoded in OFC neurons. Shaded rectangle corresponds to non-significant fraction of neurons ($p > 0.05$, n = 76 (trial-initiation), n = 87 (stimulus-offset), n = 78 (choice))

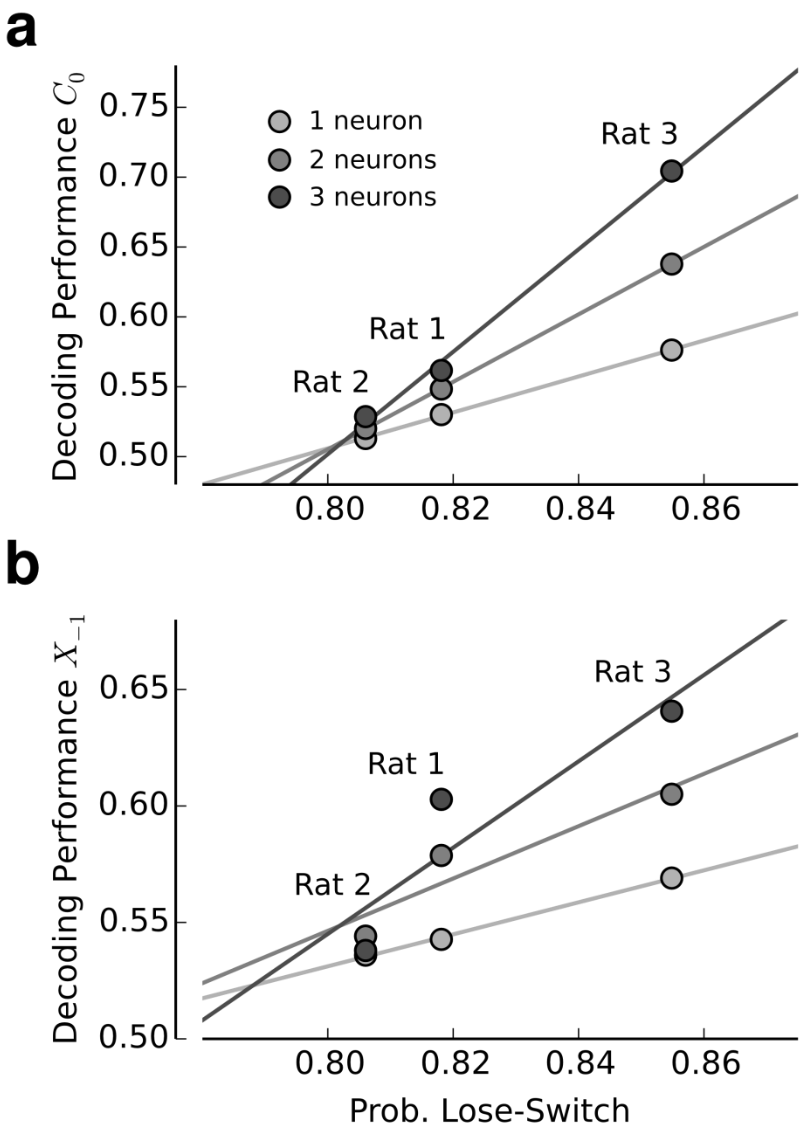

Supplementary Figure 7

Animals that have a higher probability of switching choice after an incorrect response also tend to have more information in OFC about both upcoming choice and second-order prior (**a**) Correlation between neuronal information about upcoming choice C_0 and lose-switch probability across rats. Correlation for all ensembles sizes is strong but not significant (mean Pearson correlation = 0.999, two-tailed permutation test, p = 0.167, n = 3, see Supplementary Methods). (**b**) Correlation between neuronal information about second-order prior X_{-1} and lose-switch probability across rats. Correlation for all ensembles sizes is strong but not significant (mean Pearson correlation = 0.947, two-tailed permutation test, p = 0.167, n = 3, see Supplementary Methods).

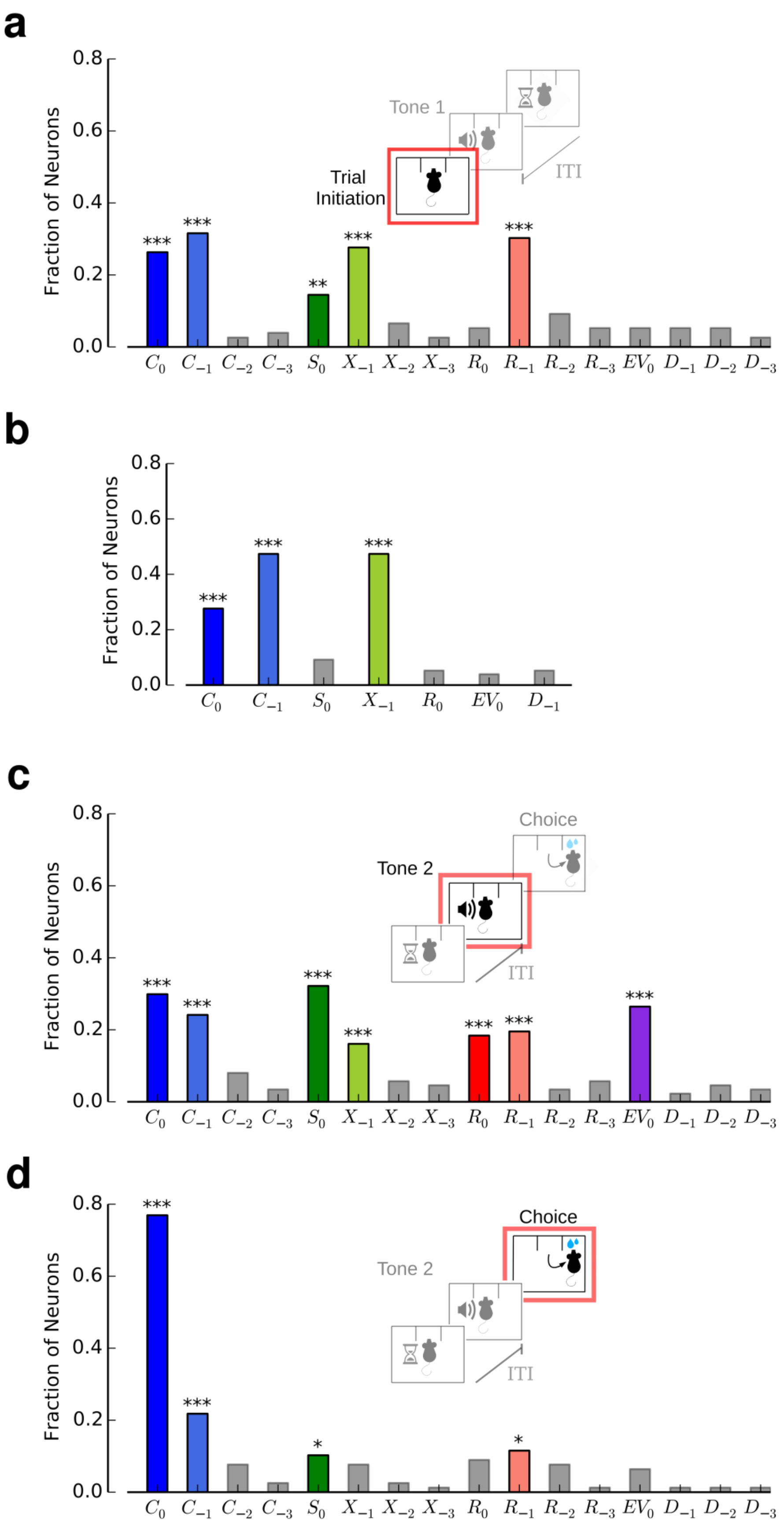
a
Fraction of Neurons
Tone 1
Trial
Initiation
ITI
b
Fraction of Neurons
c
Fraction of Neurons
Choice
Tone 2
ITI
d
Fraction of Neurons
Choice
Tone 2
ITI

Supplementary Figure 8

Using a linear regression instead of a GLM on the data presented in Fig 4 and Supplementary Fig 4. (**a-d**) Linear regression-based analysis gives virtually identical results to those in Figs. 4 and Supplementary Fig. 4 obtained from a GLM analysis. Panels a, c, and d correspond to Fig. 4a-c. Panel b corresponds to Supplementary Fig. 4. * = $p < 0.05$, ** = $p < 0.01$, *** = $p < 0.001$ (n = 76 for panel a and b, n = 87 for panel c and n = 78 for panel d).

Supplementary Methods

Presentation of acoustic stimuli

The protocols of stimulation were controlled through MATLAB, a National Instrument card (BNC-2110), and a breakout box (FS 300 kHz). Sound triggers had microsecond precision. Sound tones were delivered through earphones (ER.6i Isola- tor, Etymotic Research), which were screwed in each recording session to the earphone holders, chronically attached to the animal skull with dental cement. The earphones were adjusted inside the ear with silicone tips with a separating distance of 1 mm from the ear canal. Similarly, sound calibration was performed inside the acoustic isolation box with a microphone (MM1, Beyerdynamic) placed 1 mm away from the earphone and using a preamplifier (USB Dual Pre, Applied Research and Technology). The sound tones had a duration of 50 ms, with an intensity of 80-dB SPL pure tones of 5,322 Hz, and 6-ms rise/fall cosine ramps.

Logistic regression of behavior

Rat's choices were classified on a trial-by-trial basis by a logistic regression (linear classifier). Classification was based on a decision variable DV: when $DV > 0$ the trial was classified as belonging to class 1 (C_0 = short choice), when $DV < 0$ the trial was classified as belonging to class 2 (C_0 = long choice). The decision variable DV was a weighted sum of the all task variables we thought might be influencing rat's behavior: $DV = \sum_{i=1}^{M} \omega_i x_i + \omega_0$, where ω_i and x_i were each task variable's contribution to the decision and its particular value ($x_i = \pm\ 1$) respectively, ω_0 was the offset term and M

was the total number of variables used for predicting rat's behavior (21 regressors in total). Logistic regression assumes that the probability of C_0 = 1 (short choice) to be the correct class given the task variables is given by:

$$p(C_0 = 1|\{x_i\}) = \sigma\left(\sum_{i=1}^{k} \omega_i x_i + \omega_0\right) \qquad (1)$$

where $\sigma(\cdot)$ is the logistic function. The set of task variables $\{x_i\}$ is $S_0, R_{-n}, D_{-n}, C_{-n}$ and X_{-n} where n is the number of trials back in time, which ranged from 1 to 5. Here R_{-n} is the reward given to the rat n trials back in time i.e. the correctness of the response (+1 correct, rewarded, -1 incorrect, non-rewarded); D_{-n} is the trial difficulty, defined on the basis of the distance between the presented ITI and the category boundary (50, 100, 450 and 500 ms, easy trial, D_{-n} = +1; 150, 200, 350 and 400 ms, difficult trial, D_{-n} = -1); C_{-n} is rat's choice (+1 short choice, -1 long choice) and X_{-n} (n-back second-order prior) is the interaction term between reward and choice, $X_{-n} = R_{-n} \times C_{-n}$. Thus, the variable X_{-n} is also binary and it takes the value $X_{-n} = 1$ when R_{-n} was correct (incorrect) and C_{-n} was short (long) and the value $X_{-n} = -1$ when R_{-n} was incorrect (correct) and C_{-n} was long (short). The variable S_0 is the stimulus category (short or long) presented to the rat on the current trial.

For most sessions, the number of trials belonging to choice short did not match the number of trials belonging to choice long, in other words, conditions were unbalanced. We addressed this problem by subsampling [1, 2], which consists in balancing the number of trials for the two classes by randomly excluding trials from the most populated class. A large imbalance can be problematic when comparing classifier's performance among data sets: if class 1 and class 2 are unbalanced, then Decoding Performance (DP) can be larger than chance (DP = 0.5) even when there is no information in any of the regressors. Subsampling was repeated 20 times. Each time the model was trained and tested by 5-fold cross validation. The reported task-variable's weight was computed as the mean absolute value of the regression coefficient associated to that variable across all recording sessions, subsampling and cross-validation iterations.

To test statistical significance of each task-variable's weight we used a permutation test that sampled the null hypothesis. For each subsampling iteration (20

iterations) we shuffled choice labels (short or long choice) and computed the task-variable weights by 5-fold cross-validation. This procedure was repeated 1,000 times. The null hypothesis distribution for each task variable's weight ω_i was the mean absolute value across recording sessions, subsampling and cross-validation iterations. We defined the probability that a particular variable did not influence the rat's behavior by the fraction of samples that fell above the estimated weight's absolute value. The reported one-tailed p-values were equal to that fraction. We preferred employing a permutation to test for significance in the regressors against more traditional methods that are based on the assumption that the residuals are Gaussian [3, 4, 5], because the residuals we observed in our data were strongly non-Gaussian. Furthermore, permutation tests are in general more conservative (lower probability of type I errors). Finally, permutation tests sample the null hypothesis while taking into account correlations in the regressors.

Surgical procedure

Recordings were obtained from three Wistar rats that were chronically implanted with tetrodes in the lateral orbital frontal cortex (lOFC) (see Fig. 2a). Animals were trained for 21 days. After 1 week of water and food ad libitum, a microdrive holding the tetrodes was implanted. To perform the surgery, anesthesia was induced using intraperitoneal injections of ketamine (60 mg/kg) and medetomidine (0.5 mg/kg). The animals were then mounted in a stereotaxic frame, and their skulls exposed. A 3-mm-diameter craniotomy was made, with its center at 1600 microns dorso-ventral, 3.7 mm anterior-posterior and 2.5 mm medium-lateral from bregma [6]. Body temperature was monitored through a rectal thermometer and maintained (36–38°C) using an electric blanket. Heart rate and blood oxygen levels were monitored. Reflexes were regularly checked during surgery to assure deep anesthesia. Other drugs were given during surgery and recovery period to prevent infection, inflammation, and as analgesia: antibiotics (enrofloxacin; 10 mg/kg sc) and topical application of neomycin and bacitracin in powder (Cicatrin), analgesic (buprenorphine; 0.05 mg/kg sc), anti-inflammatory (methylprednisolone; 10 mg/kg ip), and atropine (0.05 mg/kg sc) to prevent secretions during surgery. Once the animals went through all experimental sessions, they were sacrificed by means of an overdose of pentobarbital (0.8 ml).

Tetrodes and microdrives

Each tetrode was made from four twisted strands of HM-L-coated 90% platinum-10% iridium wire of 17 µm diameter (California Fine Wire, Grover Beach, CA). Gold plating decreased their impedance to ca. 300–500 KΩ. Four tetrodes were held by a cannula attached to a microdrive supplied by Axona (St. Albans, UK). This microdrive allowed for dorsal to ventral tetrode movement to search for new units. Microdrives were attached to the skull with dental cement and seven stainless steel screws. The OFC was reached by vertical descent, and the tetrodes were lowered 1600 microns during the surgery. Vertical descent performed after surgery was of 50 microns/day until the OFC was reached [6]. This depth estimation was verified on by histological reconstruction of the electrode's tracks (see Fig. 2a).

Electrophysiological recordings from awake, freely moving rats

During the training period, animals lived in large cages of 28 x 42 x 30 cm (Charles River) in a rich environment, under a 12:12-h light-dark cycle, and with food ad libitum and water restriction. Before training and after 1 week of postoperative recovery period, the animals were accustomed to the recording chamber. The electrode wires were AC-coupled to unity-gain buffer amplifiers. Lightweight hearing aid wires (2–3 m) connected these to a preamplifier (gain of 1.000), and to the filters and amplifiers of the recording system (Axona, St. Albans, UK). Signals were amplified (x 15,000–40,000), high-pass filtered (360 Hz), and acquired using software from Axona (St. Albans, UK). Each channel was continuously monitored at a sampling rate of 48 kHz. Action potentials were stored as 50 points per channel (1 ms; 200 µs prethreshold and 800 µs postthreshold) whenever the signal from any of the pre-specified recording channels exceeded a threshold set by the experimenter for subsequent offline spike sorting analysis. Data were excluded if any drift was detected. Before each experimental session, tetrodes were screened for neuronal activity. Once spikes could be well isolated from background noise, the experimental protocol started.

Experimental setup

The recordings were performed inside a black acrylic box of dimensions 22 x 25.5 x 35 cm. This box was placed inside two wooden boxes placed one inside the other. Between each box, two isolating foam rubbers (4 and 2 cm thick) were placed to soundproof for low and high frequencies. A wooden cover and soundproof foams closed the entire recording chamber, with only a hole to allow the entry of a recording wire (2 mm thick) connected to the preamplifier. Water valves were placed outside the recording chamber. The animals poked their noses into three different sockets (2 cm wide and separated by 3 cm each, and with no cover in the top part to avoid being hit by the microdrive). Recordings were obtained in darkness, and the experiment was filmed with an infrared camera placed above the recording chamber.

Correlation between behavior and neuronal activity across rats

We correlated rats' probability of switching choice after an incorrect response (lose-switch probability) with the Decoding Performance of both upcoming choice C_0 and second-order prior X_{-1} calculated during the trial-initiation period across rats. Three different sizes of neuronal ensembles were used for this analysis: single units, pairs and triplets of neurons. The reported DPs for each rat were the mean DP across all the groups of simultaneously recorded neurons of a given size for that particular animal. Significance was tested using a permutation test that sampled the null hypothesis. For each ensemble size we shuffled the probability lose-switch vector and the Decoding Performance vector. This process gave us a total of thirty-six possible combinations (3! × 3!). We defined the probability that there was no correlation between the two vectors by the fraction of samples that fell above the real correlation value. None was above the real correlation value but six samples were equal. Therefore, we considered that out of these six, three were above and three were below the real correlation value. The reported two-tailed p-values were twice that fraction.

Power Analysis

To address whether the sample size used in this study was sufficiently large, we performed a power analysis. The power (π) in hypothesis testing is defined as the probability of correctly rejecting the null hypothesis (when it is false). It is related to the β, the probability of a type II error, through the equality π = 1- β.

We first calculated the statistical power of the fraction of neurons that had their firing rate significantly modulated by each one of the reported regressors. Because significance for each neuron and regressor was calculated using a Binomial test (one-tailed), the power analysis was calculated using a Binomial distribution as well. First, we calculated the threshold on the fraction of neurons that would make us reject the null hypothesis when it is true. This value was the smallest fraction of neurons that fulfilled $p \leq 0.05$ for a one-tailed Binomial test. As this quantity only depends on the total number of neurons used in the Binomial test, it is the same across all regressors. We calculated the statistical power of the fraction of neurons encoding for a particular regressor as the probability of sampling a largest or equal value than the threshold from a binomial distribution, with probability of success defined as the actual fraction of neurons reported in the study. For a fixed probability of type I error ($\alpha = 0.05$) the statistical power increases with the strength of the result itself (reported fraction of neurons) and the number of samples used in the Binomial test (number of neurons). In other words, the probability of making a type II error when inferring the significance of that result decreases if these quantities increase.

The same procedure was applied to the statistical power analysis associated with the number of rats. Every rat that showed results in favor of the hypothesis was considered a success, and otherwise it was a failure. Because all rats in the study belonged to the success class (our main results are consistent across rats) we calculated the power if an additional rat without significant results (belonging to failure class) was added to the study.

We considered a result had sufficient statistical power when it exceeded the standard threshold [7] $\pi = 0.80$. For the number of single units (n = 76), the statistical power of the fraction of neurons encoding C_0 before stimulus presentation was $\pi = 0.9996$, and $\pi = 0.9947$ for X_{-1}. Thus, the two central results of our study are statistically rather solid.

Regarding the number of rats, we found significant results consistently across all rats. If we included a forth rat with negative results the power of our conclusions would be $\pi = 0.95$. Thus, despite the small sample size, the strength of the results supported sufficient statistical power.

Supplementary References

1. Hastie, T., Tibshirani, R. and Friedman, J. The Elements of Statistical Learning. Springer, NY (2001).

2. He, H., and Garcia E.A. Learning for imbalanced data. *IEEE Trans. Knowledge and Data Engineering* **21**, 1263-1284 (2009).

3. Sul, J.H., Kim, H., Huh, N., Lee, D. & Jung, M.W. Distinct roles of rodent orbitofrontal and medial prefrontal cortex in decision making. *Neuron* **66**, 449-460 (2010).

4. Genovesio, A., Tsujimoto, S., Navarra, G., Falcone, R. & Wise, S.P. Autonomous encoding of irrelevant goals and outcomes by prefrontal cortex neurons. *J Neurosci* **34**, 1970-1978 (2014).

5. Donahue, C.H. & Lee, D. Dynamic routing of task-relevant signals for decision making in dorsolateral prefrontal cortex. *Nat Neurosci* **18**, 295-301 (2015).

6. Paxinos, G., and Watson, C. The rat brain in stereotaxic coordinates. San Diego, CA: Academic (1998).

7. Baussell Barker, R., and Yu-Fang, Li. Power Analysis for Experimental Research: A Practical Guide for the Biological, Medical and Social Sciences. Cambridge University Press (2002).

8. Abolafia, J.M., Martinez-Garcia, M., Deco, G., and Sanchez-Vives, M.V. Variability and information content in auditory cortex spike trains during an interval-discrimination task. *J. Neurophysiol.* **110**, 2163–2174 (2013).
